# The molecular subtyping and precision medicine in triple-negative breast cancer---based on Fudan TNBC classification

**DOI:** 10.1186/s12935-024-03261-0

**Published:** 2024-03-30

**Authors:** Lijuan Weng, Jianliang Zhou, Shenchao Guo, Nong Xu, Ruishuang Ma

**Affiliations:** 1 Department of Medical Oncology, The First Affiliated Hospital of Zhejiang University, Hangzhou, China; 2 Department of Radiotherapy and Chemotherapy, The First Affiliated Hospital of Ningbo University, Ningbo, China

**Keywords:** Triple-negative breast cancer, Molecular subtyping, Precision medicine

## Abstract

Triple-negative breast cancer (TNBC) is widely recognized as the most aggressive form of breast cancer, occurring more frequently in younger patients and characterized by high heterogeneity, early distant metastases and poor prognosis. Multiple treatment options have failed to achieve the expected therapeutic effects due to the lack of clear molecular targets. Based on genomics, transcriptomics and metabolomics, the multi-omics analysis further clarifies TNBC subtyping, which provides a greater understanding of tumour heterogeneity and targeted therapy sensitivity. For instance, the luminal androgen receptor subtype (LAR) exhibits responsiveness to anti-AR therapy, and the basal-like immune-suppressed subtype (BLIS) tends to benefit from poly (ADP-ribose) polymerase inhibitors (PARPis) and anti-angiogenic therapy. The efficacy of multi-dimensional combination therapy holds immense importance in guiding personalized and precision medicine for TNBC. This review offers a systematic overview of recent FuDan TNBC molecular subtyping and its role in the instruction of clinical precision therapy.

## Background

Triple-negative breast cancer (TNBC) is characterized by the absence of estrogen receptor (ER), progesterone receptor (PR) and human epidermal growth factor receptor-2 (HER-2), accounting for 12 − 17% of all breast cancer cases [1]. The high heterogeneity and lack of well-defined molecular targets in TNBC restrict the effectiveness of targeted therapies observed in non-selective clinical trials. Therefore, it is imperative to delineate the molecular biological characteristics of various subtypes within TNBC. With the continuous expansion of the TNBC population, multiomics-based TNBC subtypes have been identified and proposed. Classic genome- and transcriptome-based TNBC subtypes are as follows: (1) luminal androgen receptor (LAR) subtype characterized by androgen receptor signalling; (2) immunomodulatory (IM) subtype with high expression of immune cell signalling and cytokine signalling; (3) basal-like immune-suppressed (BLIS) subtype characterized by upregulation of cell cycle processes, activation of DNA repair mechanisms, and downregulation of immune response genes; and (4) mesenchymal-like (MES) subtype characterized by upregulation of the JAK/STAT3 signalling pathway. The recent categorization of TNBC subtypes has yielded significant revelations regarding tumour heterogeneity, thereby facilitating the exploration of alternative approaches to existing targeted therapies exhibiting limited efficacy and the identification of novel targets for TNBC treatment.

## Genome- and transcriptome-based TNBC subtypes

Alterations in the genomic and transcriptomic profile of TNBC could contribute to specific intracellular processes, reflecting the intrinsic biological characteristics of tumours and driving the growth of tumour cells. In 2011, Lehmann et al. preliminarily mapped the molecular subtyping of TNBC through gene expression profile characteristics and proposed for the first time to divide TNBC into six molecular subtypes [2]: basal-like (BL1 and BL2), immunomodulatory (IM), mesenchymal (M), mesenchymal stem-like (MSL), and luminal androgen receptor (LAR). In a subsequent study conducted in 2016, the research team discovered that the IM and MSL subtypes were derived from tumour-infiltrating lymphocytes (TILs) and stromal cells, respectively. As a result, the classification was revised to encompass four subtypes: BL-1, BL-2, M, and LAR [3]. This classification has significant implications for subsequent research on TNBC. However, the methodology employed for subtyping in this study is too single, could not accurately distinguish between BL-1 and BL-2 [4], and fails to provide a comprehensive representation of the genomic characteristics of TNBC. In 2015, Burstein et al. analyzed 198 cases of TNBC using whole-genome sequencing and divided TNBC into four subtypes [5]: luminal androgen receptor (LAR), mesenchymal (MES), basal-like immunosuppressed (BLIS), and basal-like immune-activated (BLIA). Compared to Lehmann subtyping, this classification can better illustrate the relationship between each subtype and prognosis. For example, regarding disease-free survival (DFS) and disease-specific survival (DSS), the prognosis is worse for the BLIS subtype and best for the BLIA subtype. The Lehmann subtyping and Burstein subtyping depict the molecular characteristics of TNBC and propose potential treatment strategies. However, the effectiveness of these subtypes has yet to be ultimately confirmed through clinical trials. In 2019, Jiang et al. analyzed 465 TNBC samples by multi-omics such as whole exome sequencing (WES), copy-number alteration (CNA), and RNA sequencing to explore the specific associations between gene expression, copy number and corresponding subtypes, and identified four subtypes for Chinese TNBC [6].

**Luminal androgen receptor subtype**. LAR refers to androgen receptor (AR) positive TNBC, and studies have demonstrated that AR expression is present in 10–35% of TNBC patients [7]. An increasing body of clinical evidence suggests the efficacy of anti-androgen therapy in this particular subtype [8]. In 2013, Gucalp et al. conducted a pioneering clinical trial on anti-androgen therapy for advanced breast cancer treatment [9]. In this study, twenty-six participants were evaluated for the primary endpoint, and the 6-month clinical benefit rate (CBR) was 19% for bicalutamide. Other Phase II clinical trials have reported promising outcomes in terms of clinical benefit for patients with advanced AR-positive TNBC following treatment with abiraterone and enzalutamide [10, 11]. The prognostic significance of AR in TNBC patients remains a topic of debate. Astvatsaturyan et al. found that AR-positive patients with TNBC were older and had a higher incidence of axillary metastases compared to AR-negative patients [12]. This study did not observe a significant difference in the mean DFS between AR-negative and AR-positive TNBC patients. However, in another study, Pistelli et al. observed that AR positivity was inversely correlated with higher Ki-67 and lympho-vascular invasion but found no association with DFS and overall survival (OS) [13]. Three prior meta-analytical studies have demonstrated a prolonged DFS in breast cancer patients with AR positivity compared to those with AR negativity. The results of the three analyses demonstrated agreement with the findings of DFS. However, Qu [14] and Wang [15] reported no correlation between AR status and OS. Conversely, Kim et al.‘s study indicated a survival benefit for AR-positive TNBC patients [16]. Results of a recent prospective study by Asano et al. corroborate that patients with AR-positive TNBC survive longer after recurrence than those with AR-negative TNBC [17]. Astvatsaturyan et al. developed a statistically significant prognostic model using a combination of epidermal growth factor receptor (EGFR) and AR [12]. Cases characterized by AR+/EGFR- exhibit a more favourable response to anti-AR therapy and prolonged DFS than cases with AR-/EGFR-, AR+/EGFR+, and AR-/EGFR+ [12]. Bi et al. discovered that the transmembrane protein TMEM25 acts as an inhibitor of monomeric EGFR-mediated STAT3 activation, thereby suppressing the progression of TNBC [18]. In conclusion, an increasing body of evidence suggests that the expression of AR is linked to a favourable prognosis. However, the precise role of the AR pathway in TNBC remains uncertain, as conflicting findings have emerged from preclinical investigations. These discrepancies among studies may be attributed to the absence of a correlation between AR expression and clinicopathologic features, or the relatively limited sample size utilized in these investigations. The prevalence of LAR is higher in the Chinese TNBC cohort compared to the TNBC cohort of the Cancer Genome Atlas (TCGA) [6]. The variations observed in the distribution of TNBC subtypes may potentially be attributed to distinct genetic backgrounds prevalent among various races or ethnic groups.

The LAR subtype exhibits a higher frequency of phosphatidylinositol 3-kinase catalytic alpha (PIK3CA) mutations [6], a more significant occurrence of ERBB2 somatic mutations, and a more frequent loss of CDKN2A compared to other subtypes. Previous studies have confirmed the effectiveness of phosphoinositide 3-kinase (PI3K) inhibitors in combination with AR inhibitors in the LAR cell model [19, 20]. It is worth noting that selective inhibitors targeting the PI3Kα subunit have shown improved effectiveness and tolerability [21], making them a prominent area of research in recent years. According to the HER2 testing guidelines for breast cancer, TNBCs are clinically HER2-negative [22]. However, it has been observed that a subset of patients with ERBB2 mutations exhibit relative activation of the ERBB2 pathway [23]. Additionally, some ERBB2 proteins can be detected in ERBB2-negative breast cells [24], indicating the potential efficacy of irreversible ERBB2 inhibitors like Neratinib [25, 26]. Abnormalities in CDKN2A have been linked to the sensitivity of LAR to CDK4/6 inhibitors or other cell cycle inhibitors [6, 27], and the resistance to CDK4/6 inhibitors is closely related to the PI3K/mTOR pathway [28]. The PI3K-Protein Kinase B (AKT)-mTOR pathway plays a crucial role in regulating the proliferation and survival of TNBC [29], indicating the effectiveness of combination CDK4/6 inhibitors with PI3K/mTOR inhibitors. Furthermore, microRNA (miRNA), a small non-coding RNA, exerts regulatory control over target genes during the post-transcriptional phase and is involved in the progression and metastasis of cancer [30–32]. Shi et al. observed a significant differential expression of 153 miRNAs between AR-positive and AR-negative breast cancer cells. These findings have important implications for using miRNA-associated pathways to inhibit AR function in breast cancer [33].

**Immunomodulatory subtype**. The IM subtype is characterized by a high expression of immune cells and cytokine signalling, as well as an enrichment of immune-activated cells and immune stimulants [34]. Additionally, there is a higher presence of both stromal and intratumoral TILs in IM patients [35, 36]. CD8 is a marker for cytotoxic T-cells, an essential component of TILs and tumour immune microenvironment [36]. After controlling for lymph node status and tumour size, IM patients exhibit a better prognosis, as concluded by several studies in the past [6, 37]. Leeha et al. conducted a retrospective cohort study [38] comprising 195 TNBC cases that were treated at a university hospital in Southern Thailand. The study employed the IHC-based subtyping method proposed by Zhao et al. [39], which was originally developed using a cohort of Chinese patients. This study revealed specific differences in the distribution of TNBC subtypes in Thai patients compared to that in the Chinese and Western populations and confirmed that the IHC-based subtype was significantly associated with OS but not DFS. Patients with the IM subtype had a better OS than those with other subtypes. In contrast to other subtypes, the IM mutation load is insignificant, rendering mutation-specific treatments ineffective. However, gene set enrichment analysis (GSEA) has confirmed the activation of the acquired immune system and IFN-γ pathway in the IM subtype. Therefore, the mechanisms by which these tumours achieve immune evasion may involve the recruitment of immunosuppressive cells or the activating of immune checkpoint molecules. Prior research has demonstrated a correlation between PD-L1 expression and heightened infiltration of CD8 + T cells in TNBC [40, 41]. High mRNA expression levels of immune checkpoint inhibitor genes, such as PD1, PDL1, CTLA4, and IDO1, were observed in the IM subtype, suggesting that these patients may benefit from immune checkpoint inhibitors (ICIs) [6, 42]. KEYNOTE-173 (a phase Ib trial evaluating neoadjuvant chemotherapy with or without pembrolizumab in early TNBC) showed that a higher combined score evaluating levels of stromal TILs and PD-L1 expression was significantly associated with higher pathologic complete response and overall response rates in patients with early-stage TNBC [43]. In a phase 1b clinical trial (NCT01633970), Atezolizumab and nab-Paclitaxel demonstrated efficacy in treating metastatic TNBC [44]. In a separate investigation, transcriptomic profiles of 107 female patients with TNBC were examined, utilizing weighted gene co-expression network analysis (WGCNA) to construct gene networks and identify co-expressed gene modules [45]. Through the application of WGCNA, it was determined that eight hub genes (BIRC3, BTN3A1, CSF2RB, GIMAP7, GZMB, HCLS1, LCP2, and SELL) associated with immunotherapy were upregulated in TNBC, and their heightened expression exhibited a positive correlation with TILs. Further exploration into the mechanisms of immune regulation may yield novel therapeutic approaches for clinical implementation in the IM subtype.

**Basal-like immune-suppressed subtype (BLIS)**. In contrast to the IM subtype, the BLIS subtype lacks immune activation, rendering it challenging to derive benefits from immune checkpoint inhibitors. BLIS is distinguished by the upregulation of cell cycle processes, heightened genomic instability, activation of DNA repair mechanisms, and the downregulation of immune response genes [6], suggesting that it might be sensitive to poly ADP-ribose polymerase (PARP) inhibitors and agents that induce DNA damage. Additionally, BLIS is frequently observed to be enriched with mutations associated with homologous recombination deficiency (HRD), with the BRCA gene playing a particularly crucial role in this pathway. Approximately 15% of patients with TNBC possess germline mutations in the BRCA1/2 genes [46]. The HRD score, proposed as a biomarker, has the potential to identify patients who may derive therapeutic benefits from DNA damage therapies, such as germline BRCA1 or BRCA2 mutation carriers [47]. Based on the HRD score, BLIS can be categorized into two subgroups: high-HRD BLIS and low-HRD BLIS subgroups. The high-HRD BLIS subgroup exhibits a more favourable prognosis and excellent responsiveness to PARP inhibitors, DNA repair inhibitors, and DNA-damaging agents. A phase III randomized multicenter trial (NCT02032823) demonstrated that adjuvant olaparib was significantly associated with increased survival free of invasive or distant disease compared to placebo for patients with high-risk TNBC and BRCA1/2 germline mutation [48]. Additionally, the I-SPY 2 trial, a phase II multicenter adaptively randomized trial, reported higher estimated rates of pathological complete response (pCR) in the triple-negative population treated with veliparib-carboplatin (51% vs. 26%) [49]. The low-HRD BLIS demonstrates an unfavourable prognosis, potentially attributed to its inclination towards whole-genome doubling, in contrast to a larger proportion of Chr9p23/Chr13q34 gene segment amplification in high-HRD BLIS [6]. Researchers are investigating further mechanisms of PARP inhibitors, including their potential to enhance medication effectiveness and overcome resistance. In their study, Muvarak et al. found that the combination of low doses of DNA methyltransferases inhibitors (DNMTis) with PARPis leads to increased efficacy of PARPis [50]. The PI3K signalling pathway is frequently altered in the LAR subtype. At the same time, other research has indicated that PI3K inhibitors hinder the expression of BRCA1/2 and enhance the sensitivity of BRCA-proficient TNBC to PARPis [51]. Furthermore, aside from its role in regulating cell proliferation and metabolism, PI3K is crucial in maintaining DNA structure stability and facilitating DNA repair [52]. Simultaneously, this study [6] employed k-means clustering and consensus clustering to categorize TNBC into six groups based on CNA peaks. It is worth noting that specific CNA peaks (Chr12p13 amplification, Chr20q13 amplification, Chr8p21 deletion) dominate in low-HRD BLIS. This observation implies that conducting experimental investigations focused on these CNA peaks could unveil potential therapeutic targets, thereby instilling renewed optimism for managing this particular subgroup.

**Mesenchymal-like subtype (MES)**. The MES subtype is positioned between LAR and the other two subtypes regarding gene spectrum mutation characteristics [6]. Consequently, it is impossible to differentiate it from other subtypes based on specific genomic features. Nonetheless, this subtype demonstrates the characteristics of breast cancer stem cells (CSCs) and the upregulation of the JAK/STAT3 signalling pathway, which play a key role in maintaining the CSCs phenotype [53]. Compared to other subtypes, the MES subtype exhibits a higher gene signature score for the activated or tyrosine-phosphorylated STAT3 (pSTAT3), indicating the potential efficacy of STAT3 inhibitors as a treatment strategy [40]. The plasticity of CSCs is intricately linked to tumour growth, invasion, recurrence, and drug resistance [54]. It has shown that CSCs could be newly generated from non-CSCs through reprogramming mechanisms, and even CSCs with different characteristics could appear [55]. Hence, it is imperative to incorporate the generation of novel CSCs into the future trajectory of CSCs targeting. Gaining further insights into the determinants that drive plasticity holds significant potential as a viable approach in combating MES-like TNBC.

## Immunohistochemistry (IHC)-based TNBC subtypes

Presently, the primary investigations concerning the molecular analysis of TNBC have predominantly concentrated on the genome and transcriptome, offering a comprehensive elucidation of the molecular biological attributes of distinct subtypes. Nevertheless, the transcriptome-based classification of TNBC poses challenges in conducting extensive clinical trials and routine clinical practice due to the exorbitant expenses associated with sequencing technology and gene expression profile analysis, intricate operational protocols, and demanding prerequisites for subsequent data interpretation capabilities. To address this issue, the Fudan University Shanghai Cancer Center (FUSCC) research team developed a practical clinical subtyping method for TNBC using IHC analysis [39]. This approach successfully categorized 210 TNBC samples into five distinct subtypes: (1) IHC-LAR subtype, characterized by the presence of AR; (2) IHC-IM subtype, characterized by the absence of AR and the presence of CD8; (3) IHC-BLIS subtype, characterized by the absence of AR, CD8, and the presence of Forkhead Box C1 (FOXC1); (4) IHC-MES subtype, characterized by the absence of AR, CD8, FOXC1, and the presence of Doublecortin Like Kinase 1 (DCLK1); and (5) IHC-uncertain subtype, characterized by the absence of AR, CD8, FOXC1, and DCLK1. Moreover, this subtyping has been validated in two separate TNBC sample cohorts, consisting of 183 and 214 samples, respectively. The careful selection of IHC markers is an essential aspect of the development of IHC-based subtyping. It is imperative that the protein expression of the chosen marker genes, as assessed by IHC, exhibits a positive correlation with their mRNA expression. Additionally, these markers should demonstrate significant distinctions among the five subtypes. CD8A and FOXC1 are identified as the highest-ranked genes In the IM and BLIS subtypes. Conversely, in the LAR subtype, AR was ranked 5th and designated as the LAR marker due to its clinical significance and practicality in IHC detection. Within the MES subtype, the gene expression of DCLK1 ranks second. However, it is worth noting that DCLK1 has been implicated in promoting metastasis in breast cancer cell lines [56] and is recognized as a stem cell marker for various cancer types [57, 58], underscoring its potential clinical relevance. Furthermore, due to its predominant presence in the cytoplasm of tumour cells and its convenient measurability, DCLK1 has been identified as the marker for the MES subtype.

Most molecular characteristics and therapeutic implications suggested in the transcriptome-based subtyping of TNBC are conserved in the corresponding IHC-based subtyping. For instance, similar to the transcriptome-based IM subtype, the IHC-IM subtype also exhibits a higher presence of both stromal and intratumoral TILs, distinguished by the infiltration of CD8 + T cells into the tumour parenchyma. Furthermore, the study mentioned above demonstrates a notable upregulation of immune checkpoint molecules, including PD1, PD-L1, CTLA4, and IDO1 [39], thereby indicating the potential efficacy of immune checkpoint blockade (ICB) in this context [59, 60]. Moreover, a positive correlation has been established between augmented lymphocytic infiltration and a more favourable prognosis in TNBC, with research indicating a significant reduction in distant recurrence rates for TNBC with every 10% increase in TILs [61]. A study [62] conducted by researchers in the Cell Journal has discovered that antigen-specific CD8 + T cells, derived from tumour-draining lymph nodes (TDLNs), undergo expansion in both in vivo and in vitro experiments when subjected to PD-1/PD-L1 ICB treatment within the tumour tissue. The elimination of this particular subset of cells results in the failure of ICB treatment, indicating the crucial role played by CD8 + T cells in the immune response against tumours. Moreover, the CD8 + T cells derived from TDLNs exhibit superior potential as adoptive T-cell therapy for enhancing anti-tumour immunity. Additional research on TNBC could potentially enhance the effectiveness of ICB treatment. In the IHC-BLIS subtype, apart from the prevalence of HRD mutations, there is also notable overexpression of vascular endothelial growth factor (VEGF). Numerous prior clinical trials have demonstrated the favourable tolerability of combining bevacizumab with taxane-based therapy in a diverse cohort of breast cancer patients, indicating that anti-angiogenic therapy may also be a viable alternative [63]. The IHC-MES subtype exhibits a diminished relapse-free survival (RFS) and a more unfavourable prognosis compared to other subtypes. Furthermore, it demonstrates heightened expression of DCLK1, an emerging therapeutic target, in addition to activating the JAK/STAT1 signalling pathway. Multivariate survival analysis indicates that the IHC-based subtyping is an independent prognostic factor for RFS, offering supplementary insights for prognostic assessment. When utilized in conjunction with conventional prognostic factors, this classification has the potential to enhance the precision of recurrence prediction. For instance, the elevated expression of specific FOXC1 marker has been correlated with tumour invasiveness and unfavourable prognosis [64, 65].

The newly proposed classification demonstrates a high level of simplicity and effectiveness. Using kappa analysis to evaluate the agreement between the IHC-based and the genome- and transcriptome-based TNBC subtypes classification, the percentage of samples classified as the same subtype by both methods was 76.7% [39]. In 2020, the team at FUSCC conducted a phase Ib/II Fudan University Shanghai Cancer Center TNBC umbrella (FUTURE) trial (NCT03805399) [66]; in this prospective study, TNBC IHC-based genomic features were utilized to categorize refractory metastatic TNBC into seven distinct precision treatment groups, to evaluate the efficacy of these targets. It is worth noting that prior clinical trials have explored various targeted treatments for TNBC; however, they did not stratify TNBC based on specific targets, potentially constraining treatment efficacy. This study [59] encompasses a cohort of 141 metastatic TNBC patients, revealing that 42 individuals (29.8%) achieved objective responses. The study findings reveal that the median values for progression-free survival (PFS) and OS are 3.4 and 10.7 months, respectively. Four of the seven treatment groups successfully met the established efficacy targets. This study initially demonstrates the potential application of TNBC subtyping in targeted therapy for refractory metastatic TNBC. IHC-based subtyping significantly streamlines the necessary testing procedures and lowers associated expenses, thereby facilitating the implementation of precision medicine for TNBC. Moreover, these results provide valuable insights for further clinical investigations.

## Metabolomics-based TNBC subtypes

### Metabolic-pathway-based subtype

In the aforementioned FUTURE trial [66], certain predetermined targets did not yield the anticipated therapeutic outcome, particularly in the LAR and BLIS subtypes. As mentioned above, abnormalities in CDKN2A have been linked to the sensitivity of LAR to CDK4/6 inhibitors or other cell cycle inhibitors. Nevertheless, the genomic analysis revealed that all TNBCs in arm B exhibited a neutral CDKN2A status, potentially diminishing the effectiveness of CDK4/6 inhibitors. The unexpected findings in the FUTURE trial could be attributed to tumour evolution and patient selection following multiline chemotherapy; another possibility is that AR may only serve as a biomarker rather than a therapeutic target [67]. At the same time, efforts should be made to seek out better targets for TNBC actively. Consequently, researchers have redirected their focus towards metabolic reprogramming. Prior research has established a correlation between metabolic dysfunction and the clinical outcomes as well as treatment responses of various cancer types [68–70]. For instance, Du et al. demonstrated that crucial regulatory factors in lipid metabolism contribute to endocrine resistance in infiltrating lobular breast cancer. However, it is worth noting that the studies mentioned above lack verification through clinical data. The alteration of energy metabolism, exemplified by the Warburg effect, is a significant characteristic of tumour cells [71]. Nevertheless, the viability of all cells is contingent upon intermediary metabolism, with glucose serving as a prominent energy source for numerous human cell types. Consequently, using small molecules to interfere with glucose metabolism poses a heightened risk of detrimental consequences on both cancerous and normal tissues. Nevertheless, there is a growing body of evidence indicating that various tumour types, as well as tumours originating from identical tissue, display unique metabolic preferences [72–74], and genetic alterations in malignant tumours can contribute to the activation of specific cellular metabolic pathways at a heightened level. The metabolic heterogeneity of tumours hinders the effectiveness of targeted metabolic drugs [75–77]. The attainment of specificity in targeting cell metabolism has been a challenge. Therefore, the clinical significance of developing treatment strategies that consider the heterogeneity of metabolic pathways is profound, as it may offer new treatment options for patients with TNBC. In 2021, the metabolite pathway enrichment analysis (MPEA) was conducted by FUSCC on a total of 465 cases in the TNBC multi-omics database, focusing on the metabolome. Through the identification of distinct metabolic features, the TNBC samples were classified into three heterogeneous metabolic-pathway-based subtypes (MPSs) [78]: MPS1, representing the lipogenic subtype (26.4%); MPS2, representing the glycolytic subtype (36.9%); and MPS3, representing the mixed subtype (36.7%). The validity of this classification, based on metabolic pathways, was confirmed through metabolomics analysis of an additional 72 samples.

**MPS1**. MPS1 is distinguished by the relative enhancement of lipid metabolism, encompassing the synthesis of fatty acids, cholesterol, and steroids, resulting in an increased abundance of myristic acid, palmitoleic acid, arachidonic acid, and other lipids. This metabolic profile renders MPS1 more susceptible to lipid synthesis inhibitors, such as the fatty acid synthase (FASN) inhibitor C75. Furthermore, the majority of MPS1 cases are comprised of the LAR subtype. As previously stated, the LAR subtype frequently displays mutations in the ERBB2 and PI3K signalling pathways [6]. Research has indicated that mutations in ERBB2 or PIK3CA can induce a lipid synthesis phenotype in untransformed epithelial cells [79, 80], suggesting a potential connection between transcriptome- and metabolome-based subtyping. Furthermore, the PI3K pathway is widely recognized as the primary signalling cascade that promotes the Warburg effect [81, 82]. Consequently, it is imperative for future investigations to delve deeper into the coordination between the PI3K pathway, lipid synthesis, and glycolysis in MPS1.

**MPS2**. MPS2 is characterized by notable upregulation of carbohydrate and nucleotide metabolism, encompassing the citric acid cycle, glycolysis, purine metabolism, and pyrimidine metabolism. Additionally, there is an abundance of intermediates in glycolysis and nucleotide metabolism. Most MPS2 cases belong to the BLIS subtype, characterized by elevated chromosomal instability and CNA. Alterations in specific genomic regions have the potential to impact glycolysis, leading to subtype-specific metabolic reprogramming. Preclinical data suggest that solute carrier family two facilitated glucose transporter member 1 (SLC2A1) and lactate dehydrogenase A (LDHA) are viable drug targets for cancer treatment. Inactivating SLC2A1 or LDHA affects the glycolytic pathway of cancer cells, ultimately leading to apoptosis in vitro and in vivo [83]. Research has demonstrated that the upregulation of glycolysis hinders the immune functions of T lymphocytes and NK cells [78], potentially resulting from lactate production. The administration of the LDHA inhibitor FX-11 leads to a notable augmentation of tumour-infiltrating CD8 + T and NK cells in the mice model, thereby potentially enhancing the responsiveness of tumours to anti-PD-1 immunotherapy in the MPS2 subtype [84]. MPS3 displays a mixed subtype of partial pathway dysregulation, aligning with its diverse phenotype, encompassing various tumour molecular subtypes.

### Metabolite-based subtype

Metabolic studies based on transcriptomic data of metabolic genes have certain limitations. For example, the abundance of metabolites might depend more on metabolic flux analysis (MFA) than on mRNA expression of metabolic genes. Additionally, critical metabolites involved in atypical metabolic pathways may be overlooked. Therefore, it is imperative to elucidate the heterogeneity of TNBC metabolism through direct assessment of metabolite abundance. In 2022, FUSCC conducted a comprehensive analysis of polar metabolomics and lipidomics in 330 TNBC samples alongside 149 paired normal breast tissues, establishing the first-ever metabolomic atlas of TNBC. Based on metabolite characteristics, TNBC can be categorized into three distinct subtypes (C1-C3), and several essential subtype-specific metabolites have been identified as potential targets for therapeutic interventions [67].

The C1 subtype, enriched with sphingolipids and almost overlaps with the LAR subtype, exhibits distinctive features such as accumulating ceramides and fatty acids. In relation to energy metabolism, it is plausible that the C1 subtype heavily relies on fatty acid metabolism. Consequently, investigating the metabolic traits of the C1 subtype could yield valuable therapeutic targets for the LAR subtype with poor efficacy in the FUTURE trial. For the metabolomic C1 subtype, sphingosine-1-phosphate (S1P), an intermediate in the ceramide synthesis pathway, exhibits promise as a therapeutic intervention. Examples of such interventions include PF-543, an inhibitor of SPHK1, and FTY-720, an FDA-approved multi-target drug targeting the ceramide pathway [85, 86]. The C2 subtype, which is enriched with oxidative and glycosylated metabolites, exhibits an upregulation of oxidative reactions and metabolites related to glycosyl transfer, such as oxidized glutathione (GSSG) and uridine diphosphate glucose (UDPG). This subtype may have a greater reliance on glutamate metabolism for energy metabolism. Targeting the biosynthesis of N-acetyl-aspartyl-glutamate (NAAG) could be a feasible therapeutic approach for addressing the metabolomic C2 subtype. The C3 subtype, characterized by hypometabolic disorder and displaying relatively minor metabolic abnormalities in comparison to healthy tissue, carries a low likelihood of recurrence.

Research has demonstrated that the BLIS subtype encompasses metabolomic C2 and C3 subtypes [67]. Machine learning techniques have proven effective in distinguishing between these two metabolic subtypes within BLIS. Given that the RFS of C2 is comparatively shorter than that of the C3 subtype, applying metabolomics-based subtyping enables a more accurate identification of high-risk groups within BLIS. Collectively, the utilization of metabolomic subtyping has enhanced the effectiveness of transcriptomic subtyping, thereby introducing novel therapeutic targets and establishing the groundwork for future personalized treatment approaches.

## Conclusions and perspectives

**Fig. 1:**
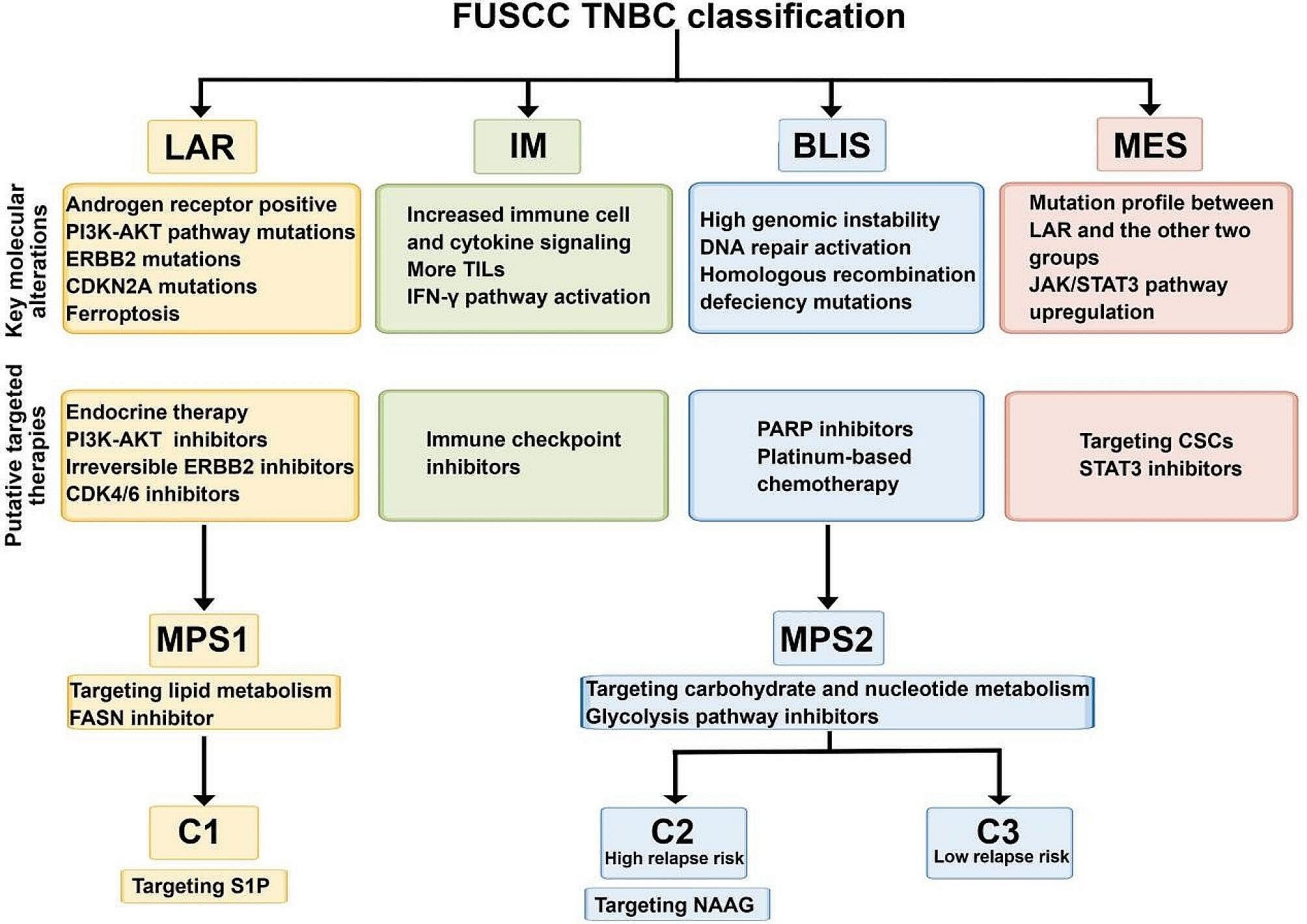
Schematic integration of molecular subtyping of triple-negative breast cancer (TNBC). Essential molecular alterations and putative targeted therapies are listed below the subtype. The LAR subtype almost overlaps with MPS1. The BLIS subtype almost overlaps with MPS2 and contains metabolomic C2 and C3 subtypes. MPS1 is highly consistent with the metabolomic C1 subtype

The aforementioned transcriptomic subtypes, metabolic-pathway-based subtypes, and metabolomic subtypes exhibit intersections: the LAR subtype almost overlaps with MPS1, and the BLIS subtype substantially overlaps with MPS2. The distribution of IM subtypes is nearly equal between MPS2 and MPS3, while the MES subtype predominantly corresponds to MPS3 [6]. Furthermore, MPS1 is highly consistent with the metabolomic C1 subtype, and MPS2/MPS3 subtypes are intricately intertwined with the metabolomic C2/C3 subtypes (Fig. 1). In the FUTURE trial, it was observed that the LAR and BLIS subtypes exhibited poor efficacy. Further investigation into their metabolic characteristics has identified metabolites S1P and NAAG as potential therapeutic targets [57]. A separate study has also confirmed the heterogeneity of ferroptosis [87], which is closely linked to metabolism in TNBC. Specifically, the LAR subtype has been found to be the most active TNBC subtype in terms of ferroptosis. The AR-driven GPX4 has been identified as a crucial factor in regulating ferroptosis, suggesting that GPX4 inhibitors could offer new treatment possibilities for LAR patients. It is worth noting that the comprehensive consideration of different subtype characteristics can optimize the precision treatment.

Presently, a significant portion of assumptions and conclusions rely heavily on laboratory data analysis. The FUTURE trial has pioneered the identification of TNBC subtypes as a potential avenue for targeted therapy in the treatment of refractory metastatic TNBC and provided substantial data and evidence to support subsequent clinical trials. Nevertheless, in this study, three of seven research groups present contradictory findings, potentially attributed to patient selection and tumour evolution following multiple rounds of chemotherapy. Consequently, numerous investigations are still required before the translation of theoretical knowledge into practical application can be achieved. Utilizing multi-omics technologies has facilitated a more comprehensive comprehension of the inherent heterogeneity in TNBC. The prominence of understanding and researching the role of non-coding RNA (ncRNA) has been increasing with the advancement of RNA sequencing technology. XU et al. discovered that long non-coding RNA (lncRNA) effectively regulates the progression of TNBC through complete interaction with miRNA at the post-transcriptional level [88]. Exploring the relationship between miRNA, lncRNA, and TNBC can offer novel perspectives for early diagnosis and treatment. Identifying novel target sites through these advancements holds potential for the selection of biomarkers, synthesis of therapeutic agents, and design of clinical trials, thereby paving the way for the exploration of more accurate and individualized treatment strategies. There is an urgent requirement for further research endeavours and prospective clinical data to elucidate the subtyping and distinctive characteristics of TNBC systematically. It is firmly believed that the implementation of precision treatment holds immense promise for significantly benefiting TNBC patients in the future.

## Data Availability

This study was based on publicly published papers. Please see Refs. for details and links to the data.

## Abbreviations

TNBC: Triple-negative breast cancer
LAR: Luminal androgen receptor
BLIS: Basal-like immune-suppressed subtype
MES: Mesenchymal-like
PARP: Poly ADP-ribose polymerase
PARPis: Poly (ADP-ribose) polymerase inhibitors
ER: Estrogen receptor
PR: Progesterone receptor
HER-2: Human epidermal growth factor receptor-2
IM: Immunomodulatory
M: Mesenchymal
MSL: Mesenchymal stem-like
WES: Whole exome sequencing
CNA: Copy-number alteration
TCGA: TNBC cohort of the Cancer Genome Atlas
PIK3CA: Phosphatidy-linositol 3-kinase catalytic alpha
PI3K: Phosphoinositide 3-kinase
AKT: Protein Kinase B
TILs: Intratumoural tumour-infiltrating lymphocytes
GSEA: Gene set enrichment analysis
WGCNA: Weighted gene co-expression network analysis
HRD: Homologous recombination deficiency
pCR: Pathological complete response
DNMTis: DNA methyltransferases inhibitors
CSCs: Cancer stem cells
pSTAT3: Tyrosine-phosphorylated STAT3
IHC: Immunohistochemistry
FUSCC: Fudan University Shanghai Cancer Center
FOXC1: Forkhead Box C1
DCLK1: Doublecortin Like Kinase 1
TDLNs: T cells, derived from tumour draining lymph nodes
VEGF: Vascular endothelial growth factor
RFS: Relapse-free survival
FUTURE: Fudan University Shanghai Cancer Center TNBC umbrella
CI: Confidence interval
EGFR: Epidermal growth factor receptor
RFS: Relapse-free survival
PFS: Progression-free survival
OS: Overall survival
CR: Complete response
PR: Partial response
SD: Stable disease
CBR: Clinical benefit rate
DFS: Disease-free survival
DSS: Disease specific survival
MPEA: Metabolite pathway enrichment analysis
MPSs: Metabolic-pathway-based subtypes
ICB: Immune checkpoint blockade
ICIs: Immune checkpoint inhibitors
SLC2A1: Glucose transporter member 1
LDHA: Lactate dehydrogenase A
FASN: Fatty acid synthase
MFA: Metabolic flux analysis
S1P: Sphingosine-1-phosphate
GSSG: Oxidized glutathione
UDPG: Uridine diphosphate glucose
NAAG: N-acetyl-aspartyl-glutamate
miRNA: MicroRNA
ncRNA: Non-coding RNA
lncRNA: Long non-coding RNA
