# Correction: The molecular subtyping and precision medicine in triple-negative breast cancer--- based on Fudan TNBC classification

**DOI:** 10.1186/s12935-025-03799-7

**Published:** 2025-04-30

**Authors:** Lijuan Weng, Jianliang Zhou, Shenchao Guo, Nong Xu, Ruishuang Ma

**Affiliations:** 1 Department of Medical Oncology, The First Affiliated Hospital of Zhejiang University, Hangzhou, China; 2 Department of Radiotherapy and Chemotherapy, Ningbo First Hospital Zhejiang University Ningbo Hospital, Ningbo, China

**Correction to: Cancer Cell International (2024) 24:120**

10.1186/s12935-024-03261-0

In this article [1], the author would like to update the second affiliation as below for funding purpose.

Incorrect affiliation:

Department of Radiotherapy and Chemotherapy, The First Affiliated Hospital of Ningbo University, Ningbo, China.

Correct affiliation:

Department of Radiotherapy and Chemotherapy, Ningbo First Hospital Zhejiang University Ningbo Hospital, Ningbo, China.
